# The beneficial effect of Zinc(II) on low-dose chemotherapeutic sensitivity involves p53 activation in wild-type p53-carrying colorectal cancer cells

**DOI:** 10.1186/s13046-015-0206-x

**Published:** 2015-08-22

**Authors:** Alessia Garufi, Valentina Ubertini, Francesca Mancini, Valerio D’Orazi, Silvia Baldari, Fabiola Moretti, Gianluca Bossi, Gabriella D’Orazi

**Affiliations:** Department of Medical, Oral and Biotechnological Sciences, University “G. d’Annunzio”, 66013 Chieti, Italy; Translational Research Department, Regina Elena National Cancer Institute, 00158 Rome, Italy; Institute of Cell Biology and Neurobiology, National Research Council of Italy (CNR), Roma, Italy; Department of Surgical Sciences, Sapienza University, 00100 Rome, Italy; Laboratory of Medical Physics and Expert Systems, Regina Elena National Cancer Institute, 00158 Rome, Italy

**Keywords:** wtp53 activation, Zinc supplementation, DNA binding, Gene expression, Colon cancer

## Abstract

**Background:**

Activation of wild-type p53 in response to genotoxic stress occurs through different mechanisms including protein conformation, posttranslational modifications, and nuclear localization, leading to DNA binding to sequence-specific promoters. Zinc ion plays a crucial role in stabilizing p53/DNA binding to induce canonical target genes. Mutant p53 proteins undergo protein misfolding that can be counteracted by zinc. However, whether zinc supplementation might have a beneficial antitumor effect in wild-type p53-carrying cells in combination with drugs, has not been addressed so far.

**Methods:**

In this study we compared the effect of two antitumor treatments: on the one hand wild-type p53-carrying colon cancer cells were treated with low and high doses of chemotherapeutic agent Adriamycin and, on the other hand, Adriamycin was used in combination with ZnCl_2_. Biochemical and molecular analyses were applied to evaluate p53 activity and biological outcomes in this setting. Finally, the effect of the different combination treatments were applied to assess tumor growth *in vivo* in tumor xenografts.

**Results:**

We found that low-dose Adriamycin did not induce p53 activation in wtp53-carrying colon cancer cells, unless in combination with ZnCl_2_. Mechanistically, ZnCl_2_ was a key determinant in inducing wtp53/DNA binding and transactivation of target genes in response to low-dose Adriamycin that used alone did not achieve such effects. Finally, *in vivo* studies, in a model of wtp53 colon cancer xenograft, show that low-dose Adriamycin did not induce tumor regression unless in combination with ZnCl_2_ that activated endogenous wtp53.

**Conclusions:**

These results provide evidence that ZnCl_2_ might be a valuable adjuvant in chemotherapeutic regimens of colorectal cancer harboring wild-type p53, able to both activate p53 and reduce the amount of drugs for antitumor purposes.

## Background

Colorectal cancer (CRC) is still the third most common cancer and the second most common cause of cancer-related death in Western countries [1]. Despite recent improvement in treating CRC, the response rate still remains low [2]. Of the patients, 30 % have advanced disease at presentation, either locally or at distant sites, so surgery is rarely a sufficient treatment. Consequently, chemotherapy alone or in combination with other agents remains the only viable therapeutic option, although the response of cancer cells to genotoxic therapies may be critically impaired by genetic alterations or drug-resistant mechanisms [3, 4].

The tumor suppressor p53 is a transcription factor that, upon DNA damage, is activated to induce target genes involved in either cancer cell growth arrest or apoptosis and its proper activation is central for efficient response to therapies aiming at tumor eradication [5]. Activation of wild-type (wt) p53 occurs in response to genotoxic stress essentially through posttranslational modifications, such as acetylation and phosphorylation, resulting in protein stabilization (by escape from proteasome-mediated degradation) and nuclear localization leading to binding to sequence-specific promoters of target genes as final outcome of its function as transcription factor [6]. TP53 includes one zinc ion (Zn2+) as an important cofactor for the proper protein folding to efficiently bind to the DNA [7]. Seminal studies in the past years showed that changes in intracellular zinc levels may inactivate wtp53 function by inducing the protein to switch to a `mutant-like' conformation with loss of DNA-binding capacity [8–10]. Interestingly, mutant p53 proteins are prone to the loss of the Zn2+ bound to the DNA binding domain (DBD) with the consequence of protein unfolding and aggregation with loss of wild-type activity [11]. Taking advantage of those findings, in recent studies we addressed the issue of mutant p53 reactivation by ZnCl_2_ or zinc complex for anticancer purposes. The combination of drug treatment and zinc restores p53 oncosuppressive activity, thus reducing the chemoresistance of mutant p53-carrying cancer cells with increased cancer cell death *in vitro* and inhibition of tumor growth *in vivo* [12–16]. These findings opened the way to the drug development of mutant p53 reactivators exploiting the effect of zinc [17, 18]. To the best of our knowledge, however, the effect of zinc supplementation on drug response of wtp53-carrying cancer cells has not been evaluated yet and, in this study, we aimed to address this theme.

We found that zinc supplementation to wtp53-carrying colon cancer cells markedly improved p53 stability/activity in response to low-dose Adriamycin (ADR) that used alone did not show p53 stabilization nor efficient cytotoxic effect. Mechanistically, we found that zinc co-treatment with low-dose ADR allowed more efficient wtp53 nuclear accumulation and DNA binding for transactivation of target genes, compared to drug treatment alone. An *in vivo* study of tumor growth in a model of wtp53 colon cancer xenograft showed that zinc treatment increased low-dose drug response with marked impairment of tumor growth. This effect was due, at least in part, to zinc-induced wtp53 activation, in agreement with the *in vitro* data. These findings demonstrate the beneficial effect of zinc supplementation in wtp53-carrying colorectal cancer cells both improving wtp53 activity and reducing the dose of chemotherapy drugs required for cytotoxic antitumor purposes.

## Materials and methods

### Ethics statement

All animals were handled in strict accordance with good animal practice as defined by the relevant national and/or local animal welfare bodies, and in accordance with the Italian and European legislation. All experimental protocol were approved by the Ethical Committee for animal research of the National Cancer Institute Regina Elena, in Rome, Italy, and by the Italian Ministry of Health, and performed in accordance with the Italian and European legislation.

### Cell culture and treatments

RKO and HCT116 human colon cancer cell lines were maintained in DMEM (Life Technology-Invitrogen), supplemented with 10 % heat-inactivated fetal bovine serum (FBS), 100 units/mL penicillin, 100 μg/mL streptomycin (Life Technology-Invitrogen), and 2 mmol/LL-glutamine in a humidified atmosphere with 5 % CO_2_ and 95 % air at 37 °C. For treatments, zinc chloride (ZnCl_2_) (SIGMA) was added to the culture medium to a final concentration of 100 μM for the indicated time points; and Adriamycin (ADR) was added to the culture medium at different concentrations, ranging between 2 and 0.1 μg/ml (3.4 and 0.17 μM, respectively). The ADR dose of 0.1 μg/ml and 1 μg/ml were considered, respectively, low and high, for the purpose of the study.

### Cell viability

Subconfluent cells were plated in triplicate in 60 mm Petri dishes and 24 h later treated with ADR or ZnCl_2_ alone or in combination for the indicated time points. Both floating and adherent cells were collected and cell viability was determined by Trypan blue exclusion by direct counting with a haemocytometer, as reported [19]. The percentage of cell viability, as blue/total cells, was assayed by scoring 200 cells per well three times.

### Western immunoblotting and immunoprecipitation

Cells were harvested from cultured dishes and total cell extracts were prepared by incubating at 4 °C for 30 min in lysis buffer [50 mM Tris–HCl (pH 7.5), 50 mM NaCl, 5 mM EDTA, 150 mM KCl, 1 mM DTT, 1 % Nonidet P-40] and a mix of protease inhibitors (Roche Diagnostic). Cell lysates were spun at 15000 g for 15 min to remove debris and collect the supernatant (total cell extracts). Protein concentration was then determined using BCA Protein Assay Kit (Biorad). Nuclear extracts were prepared essentially as described [20]. Briefly, cells were suspended in hypotonic buffer [10 mM HEPES, pH 7.9, 10 mM KCl, 0.1 mM EDTA, 0.1 mM EGTA] and placed on ice for 15 min. Nonidet P-40 was added to a final concentration of 0.5 %. Cells were spun top speed for 30 s and the supernatant (cytoplasmic fraction) was discharged. The remaining pellet was washed with hypotonic buffer, resuspended in lysis buffer as above and spun at 15000 g for 15 min to remove debris and collect the supernatant (nuclear fraction). Total or nuclear cell lysates (10 to 40 μg protein/lane) were resolved by 9–18 % SDS-polyacrylamide gel electrophoresis. For immunoprecipitation analyses, total cell extracts were immunoprecipitated with anti-p53 antibodies (Ab-DO1, Santa Cruz Biotechnology) preadsorbed to protein G–agarose (Pierce) for 2 h at 4 °C. Immunocomplexes were collected by centrifugation, separated by 9 % SDS–PAGE. Proteins were transferred to a polyvinylidene difluoride (PVDF) membrane (Millipore). The blotted membranes were blocked with 5 % skim milk for 1 h and then were incubated with the following primary antibodies: monoclonal anti-poly(ADP-ribose) polymerase cleaved form (PARP, BD Pharmingen, CA, USA), monoclonal anti-p53 (Ab-DO1), polyclonal antip53 (FL393), polyclonal anti-p21, monoclonal anti-MDM2 (Ab1, Santa Cruz), rabbit polyclonal anti-lamin A (all from Santa Cruz Biotechnology), rabbit polyclonal phospho-Ser46 and phospho-Ser15 (Cell Signalling and Santa Cruz), purified mouse anti-phospho-Histone H2AX (Ser139) (Millipore, clone JBW301; kindly provided by S. Soddu, Regina Elena National cancer Institute, Rome, Italy) and monoclonal anti-β-actin (Calbiochem). The immunoreactive bands were visualized by enhanced chemoluminescence (ECL; Amersham) using horseradish peroxidase-conjugated IgG secondary antibodies (BioRad).

### RNA extraction and semi-quantitative reverse transcription (RT)-PCR analysis

Cells and tumors were harvested in TRIzol Reagent (Invitrogen) and total RNA was isolated following the manufacturer’s instructions. Semi-quantitative RT-PCR was performed essentially as previously described [12]. Briefly, The first strand cDNA was synthesized from 2 μg of total RNA with MuLV reverse transcriptase kit (Applied Biosystems). Semi-quantitative Reverse-Transcribed (RT)-PCR was carried out by using Hot-Master Taq polymerase (Eppendorf) with 2 μl cDNA reaction and genes specific oligonucleotides under conditions of linear amplification. PCR products were resolved on 2 % agarose gels and visualized by ethidium bromide staining using UV light. The housekeeping 28S gene, used as internal standard, was amplified from the same cDNA reaction mixture. Densitometric analysis was applied to quantify mRNA levels compared to control gene expression.

### Chromatin immunoprecipitation (ChIP)

ChIP analysis was carried out essentially as previously described [20]. Briefly, protein complexes were cross-linked to DNA in living cells by adding formaldehyde directly to the cell culture medium at 1 % final concentration for 10 min at room temperature and then inactivated by the addition of 125 mM glycine. Chromatin extracts containing DNA fragments with an average size of 500 bp were incubated overnight at 4 °C with skim milk, shaking using polyclonal anti-p53 antibody (FL393, Santa Cruz Biotechnology). Before use, protein G (Pierce) was blocked with 1 μg/μl sheared herring sperm DNA and 1 μg/μl BSA for 3 h at 4 °C and then incubated with chromatin and antibody for 2 h at 4 °C. PCR was performed with Hot-Master Taq (Eppendorf) using 2 μl of immunoprecipitated DNA and promoter-specific primers spanning p53 binding sites. Immunoprecipitation with non-specific immunoglobulins (IgG, Santa Cruz Biotechnology) was performed as negative controls. The amount of precipitated chromatin measured in each PCR was normalized with the amount of chromatin present in the input of each immunoprecipitation. PCR products were resolved on 2 % agarose gel and visualized with ethidium bromide staining using UV light.

### *In vivo* tumor growth

*In vivo* experiments were performed essentially as described [12]. Six-week-old CD-1 nude (nu/nu) mice (Charles River Laboratories) were used for *in vivo* studies. They were housed in specific pathogen-free conditions and fed standard cow pellets and water ad libitum. Studies were performed in accordance with the National Cancer Institute Regina Elena standard guidelines for animal care and all experimental protocol were approved by the Ethical Committee for animal research of the National Cancer Institute Regina Elena, in Rome, Italy, and by the Italian Ministry of Health, and performed in accordance with the Italian and European legislation. Solid tumors were obtained by injecting i.m. on the flank of each mouse 4x10^6^ viable RKO cells suspended in 0.1 mL PBS. The mice were examined every day after injection until tumors reached approximately 300 mm^3^ volume (about 7 days from injection). Mice were then randomized in four groups (6–8 mice/group) as follow: 1) PBS (Mock), 2) ADR, 3) ZnCl_2_, 4) ADR plus ZnCl_2_. At day 7, mice were injected with ADR (1 mg/kg body weight) i.p.; subsequently, ZnCl_2_ (10 mg zinc/kg body weight) was administrated once daily by oral administration, over the course of about two weeks. Tumor dimensions were measured every other day and their volumes were calculated from caliper measurements of two orthogonal diameters (x and y, larger and smaller diameters, respectively) by using the formula, volume = xy^2^/2. The antitumor effect of the combination treatment, ZnCl_2_ + ADR, was evaluated by comparing the relative tumor size with tumors treated with ADR only or ZnCl_2_ only. The experiment was repeated twice with similar results.

### Statistical analyses

All experiment unless differently indicated were performed at least three times. Results are expressed as values of mean ± standard deviation (SD). Paired *t*-test (two-tailed) was used for assessing the statistical significance of the differences between treatment groups (*P* = 0.05).

## Results

### ZnCl_2_ increases p53 protein levels in the presence of low-dose ADR

**Fig. 1:**
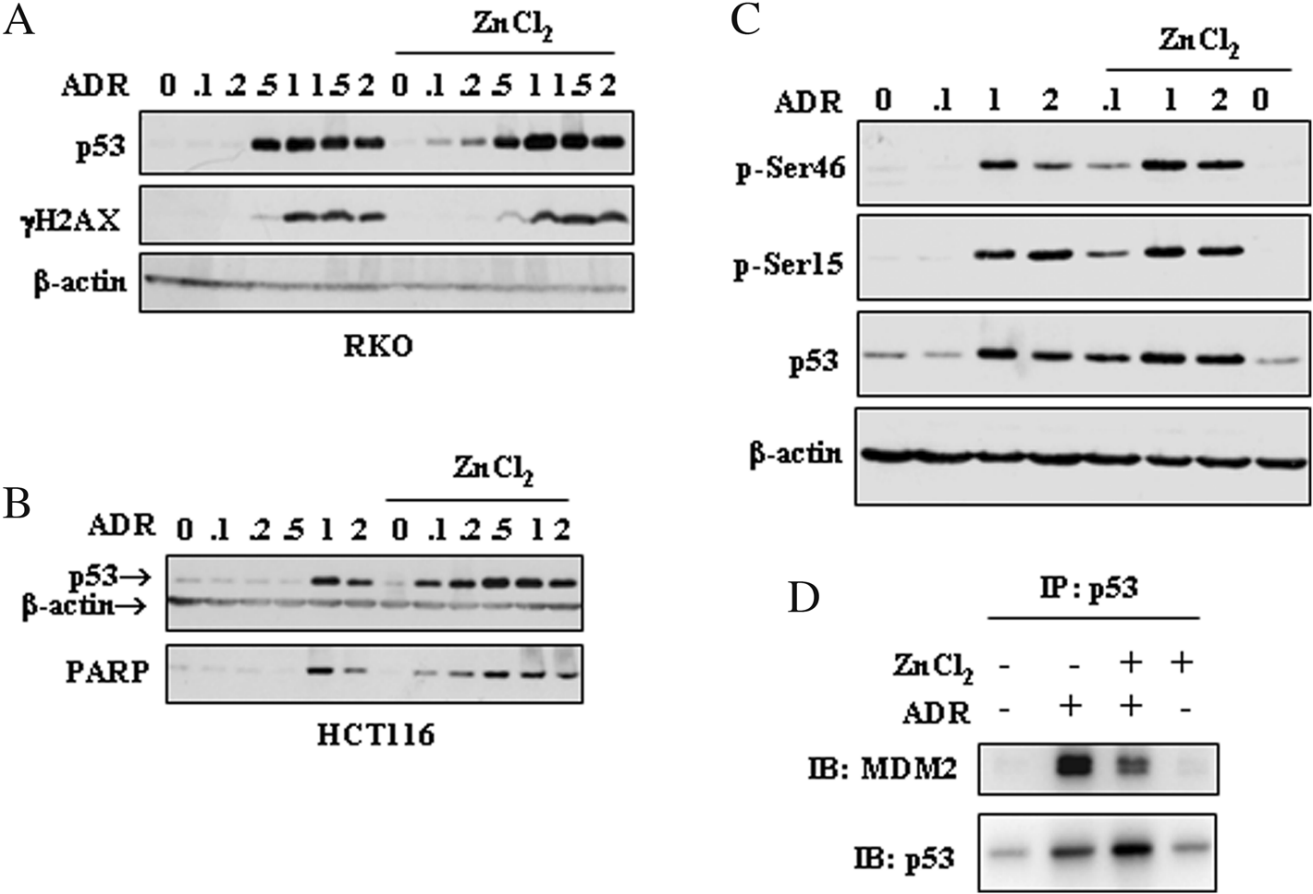
ZnCl_2_ increases the low-dose ADR-induced p53 stabilization in colon cancer cells. (**a**) RKO and (**b**) HCT116, plated under the same confluence condition, were treated with increasing doses (0.1 to 2 μg/ml) of ADR in the presence or absence of ZnCl_2_ (100 μM), for 24 h. Equal amounts of total cell lysates were subjected to immunoblot analysis for the detection of the expression levels of p53, γH2AX, and PARP (cleaved form). The samples derive from the same experiment and gels/blots were processed in parallel. (**c**) The phosphorylation of p53 at Ser15 and Ser46 was detected in HCT116 treated with ADR (0.1-1-2 μg/ml) in the presence or absence of ZnCl_2_ (100 μM) for 24 h, by western blotting. Anti-β-actin was used as protein loading control. The samples derive from the same experiment and gels/blots were processed in parallel. The gels have been run under the same experimental conditions and one representative set of blot from three independent experiments, all generating similar results, is shown here. (**d**) HCT116 cells were treated with ADR (0.2 μg/ml) and ZnCl_2_ (100 μM) for 24 h. After treatments, total cell extracts were immunoprecipitated with anti-p53 antibody. Western blot analysis was performed with anti-p53 and anti-MDM2 antibodies. IP: immunoprecipitation. IB: immunoblotting

To evaluate the *in vitro* effect of zinc supplementation on wtp53 activation in response to drug, RKO and HCT116 colon cancer cells, carrying wtp53, were treated with the chemotherapeutic drug ADR that has been widely used for treatment of a broad spectrum of cancers [21], although it can induce harmful dose-dependent effects in different sites [22, 23]. We compared two different treatment strategies: on the one hand colon cancer cells were incubated with increasing concentrations of ADR, ranging between 2 and 0.1 μg/ml (3.4 and 0.17 μM, respectively) for 24 h; on the other hand, dose–response ADR incubation was carried out in the presence of ZnCl_2_. We first analysed p53 protein levels by western immunoblotting of total cell extracts. We found that ADR alone induced p53 expression starting at 0.5 μg/ml in RKO cells (Fig. 1a) and at 1 μg/ml in HCT116 cells (Fig. 1b); interestingly, ZnCl_2_ induced p53 stabilization in combination with ADR at dose as low as 0.1 μg/ml, in both cell lines (Fig. 1a, b). Intracellular amount of p53 is regulated by phosphorylation at different sites within the protein to enhance stability and activity as transcription factor [6], therefore, we investigated p53 phosphorylation in this setting. We found that p53 was phosphorylated in serine (Ser) 15 and 46 by ADR alone at 1 μg/ml dose, while ZnCl_2_ co-treatment induced p53 phosphorylation at 0.1 μg/ml ADR, and likewise protein stabilization (Fig. 1c). To evaluate whether p53 stabilization following ZnCl_2_ co-treatment with low-dose ADR could depend on increased DNA damage response (DDR), we analysed histone H2AX phosphorylation. The phosphorylation of the subtype of histone H2A, called H2AX, in the position of Ser139 producing γH2AX, occurs in response to formation of double strand brakes (DSB) and the analysis of γH2AX expression can be used to detect the genotoxic effect of different anticancer agents [24]. Western blot analysis shows that γH2AX phosphorylation did not significantly change between the two treatments (i.e., ADR alone or in combination with ZnCl_2_) (Fig. 1a), suggesting that ZnCl_2_ was not increasing DNA damage and that, consequently, was not the mechanism responsible for higher p53 stabilization. The phosphorylation of p53 at N-terminal Ser15 and Ser46 has been shown to contribute to p53 stability by preventing the p53 negative regulator MDM2 from degrading it [25, 26]. We then investigated the p53/MDM2 interaction. We treated HCT116 cells with low-dose ADR alone or in combination with ZnCl_2_ and, after treatments, total cell extracts were immunoprecipitated with anti-p53 antibody. Normalization of total cell extracts was applied in order to have approximately the same amount of p53 immunoprecipitated in the ADR alone and ADR/ZnCl_2_ combination. As shown in Fig. 1d, MDM2 binding to p53 following ADR treatment was significantly reduced by ZnCl_2_ co-treatment (*P* = 0.003). This effect is in line with previous studies showing that zinc neutralizes the ubiquitine ligase activity of MDM2 restoring p53 activity [27, 28].

**Fig. 2:**
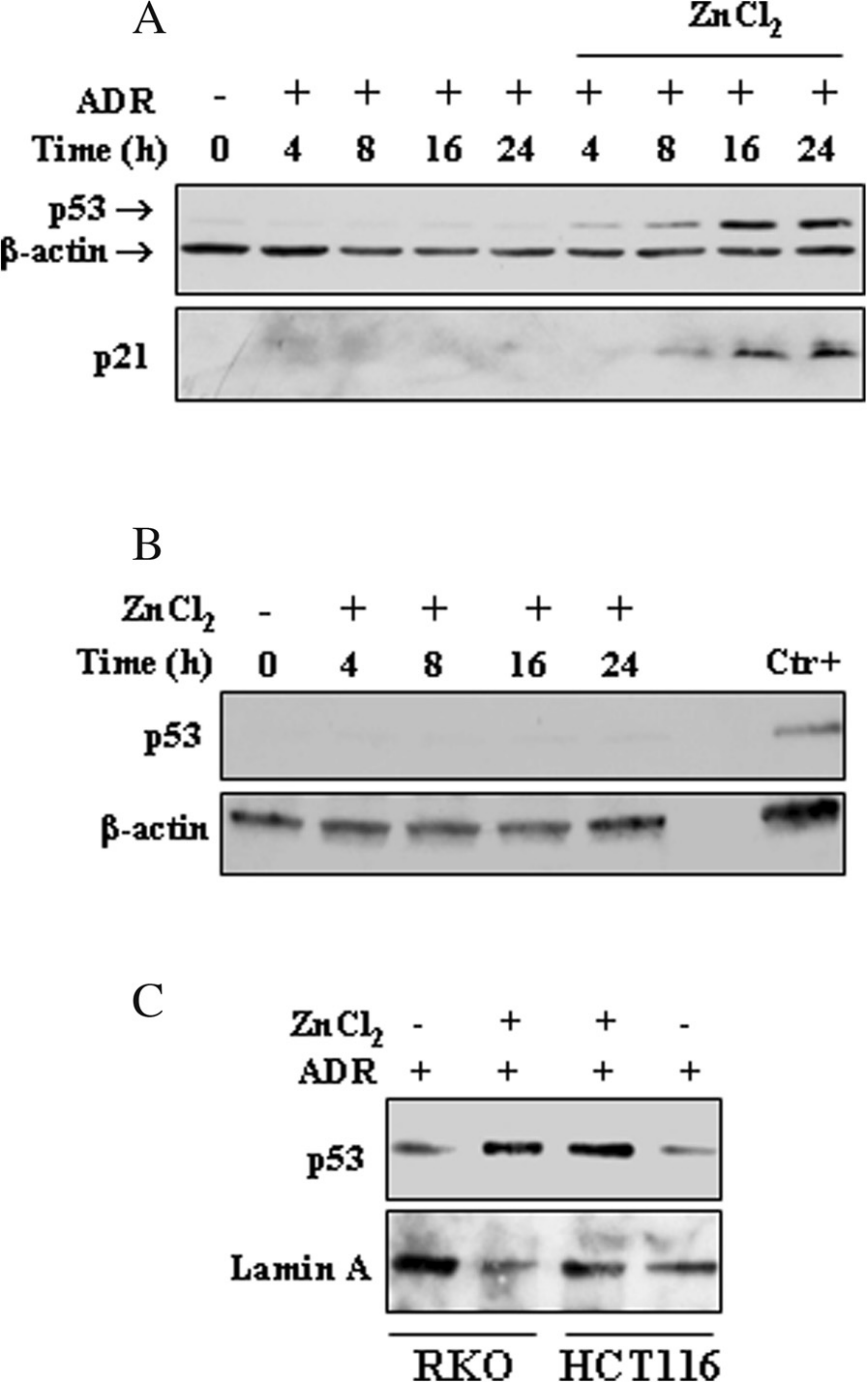
ZnCl_2_ induces p53 nuclear accumulation and activation in the presence of low-dose ADR. (**a**) HCT116 were treated with low-dose ADR (0.1 μg/ml) in the presence or absence of ZnCl_2_ (100 μM), for 4-8-16-24 h. Equal amounts of total cell lysates were subjected to immunoblot analysis for the detection of the expression levels of p53 and p21. Anti-β-actin was used as protein loading control. The samples derive from the same experiment and gels/blots were processed in parallel. One representative set of blot from three independent experiments, all generating similar results, is shown here. (**b**) RKO were treated with ZnCl_2_ (100 μM), for 4-8-16-24 h and the expression levels of p53 was detected by western blotting. A positive control for p53 expression in the same cells, is included (ADR 2 μg/ml for 16 h). Anti-β-actin was used as protein loading control. (**c**) RKO and HCT116 were treated with low-dose ADR (0.1 μg/ml) in the presence or absence of ZnCl_2_ (100 μM), 8 h. Equal amounts of nuclear extracts were separated by SDS-PAGE and p53 levels detected by western blotting. Anti-Lamin A was used as protein loading control. The gels have been run under the same experimental conditions and one representative set of blot from three independent experiments, all generating similar results, is shown here

Time-course experiment shows that while 0.1 μg/ml ADR alone did not induce p53 stabilization during the 24 h treatment, ZnCl_2_ co-treatment increased p53 protein levels beginning at 4 h post-treatment (Fig. 2a); parallel to p53 stabilization, the downstream effector p21 WAF1/Cip1 (p21) was also upregulated (Fig. 2a), suggestive of p53 activation. ZnCl_2_ treatment alone did not induce p53 levels (Fig. 2b), indicative of zinc effectiveness for p53 activation specifically in combination with drug. For PARP expression in Fig. 1b see below. Finally, we examined p53 nuclear localization, which, along with p53 stability is at the basis of p53 transcriptional activity [6]. Western blotting of nuclear extracts show that low-dose ADR (0.1 μg/ml) did not efficiently induce p53 nuclear accumulation unless in combination with ZnCl_2_, in both cell lines (Fig. 2c). Altogether, these results demonstrate that zinc supplementation could improve wtp53 stability, nuclear accumulation, and activation following co-treatment with low-dose ADR that used alone did not show such effects, at least in our experimental conditions.

### ZnCl_2_ improves the DNA binding as well as transcriptional activity of p53 in the presence of low-dose ADR

**Fig. 3:**
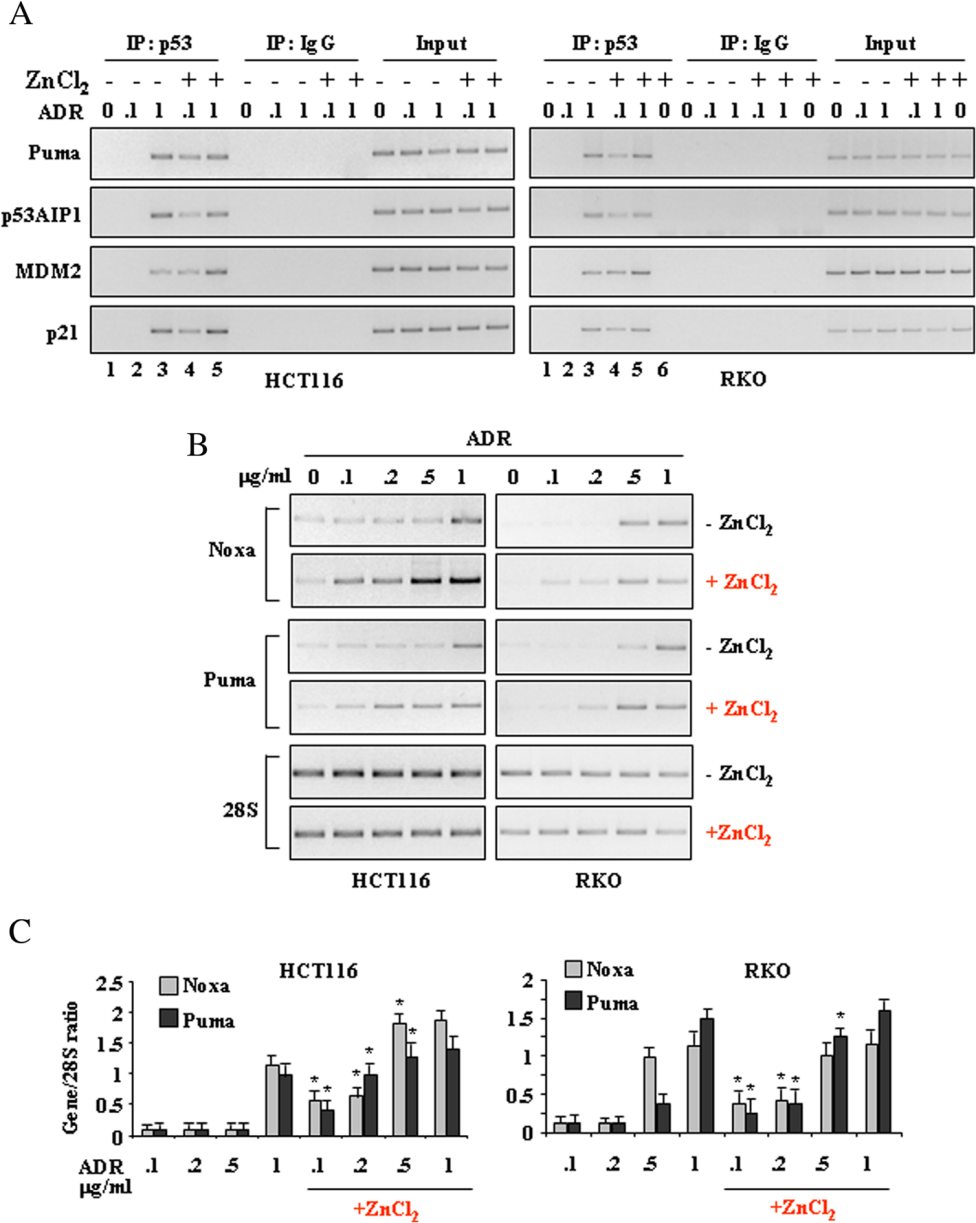
ZnCl_2_ induces p53/DNA binding and transactivation activities in the presence of low-dose ADR. **a** HCT116 (*left panel*) and RKO cells (*right panel*) (4x10^6^) were plated in 150 mm dish and the day after treated with ADR (0.1-1 μg/ml) and ZnCl_2_ (100 μM) for 16 h before being assayed for ChIP analysis with anti-p53 antibody. PCR analyses were performed on the immunoprecipitated DNA using primers specific for p53 target genes. A sample representing linear amplification of the total chromatin (Input) was included as control. Additional controls included immunoprecipitation performed with nonspecific immunoglobulins (IP: IgG). One representative experiment, out of two independent experiments generating similar results, is shown here. **b** HCT116 and RKO cells were treated with ADR (0.1-0.2-0.5-1 μg/ml) in the presence or absence of ZnCl_2_ (100 μM) for 16 h before total mRNAs were reverse transcribed for analysis of p53 apoptotic target genes (Noxa and Puma) expression, using RT-PCR. 28S was used as a control for efficiency of RNA extraction and transcription. One representative experiment, out of two independent experiments with similar results, is shown here. **c** Densitometric analysis of gene expression as shown in (**b**) in HCT116 (*left panel*) and RKO (*right panel*) cells was plotted as expression ratio to 28S. The data represent the mean of two independent experiments ± S.D. Paired student’s *t* test was used for the statistical analysis of the normalized gene expression (Gene/28S ratios) at different ADR doses, with or without ZnCl_2_ (*: *P* < 0.01)

We then evaluated whether p53 stabilization matched the induction of its transcriptional activity. To this aim, *in vivo* p53-DNA binding activity was analysed by chromatin immunoprecipitation (ChIP) technique. RKO and HCT116 cells were treated with low (0.1 μg/ml) and high dose (1 μg/ml) ADR for 16 h in the presence or absence of ZnCl_2_ and endogenous p53 immunoprecipitated with polyclonal anti-p53 antibody. The amount of co-precipitated p53-binding elements in target promoters was determined by PCR. The results show that low-dose ADR did not recruit p53 onto canonical target promoters, such as *Puma*, *p53AIP1*, *MDM2*, and *p21*, unless in combination with ZnCl_2_, in both HCT116 (*left panel*) and RKO cells (*right panel*) (Fig. 3a, compare lanes 2 with lanes 4). As a control we used the high dose ADR (1 μg/ml) that was efficient to induce p53 binding to target promoters (Fig. 3a, compare lanes 3 with lanes 2) and such recruitment was not further increased by ZnCl_2_ (Fig. 3a, compare lanes 3 with lanes 6), suggesting that p53 was fully activated by the high dose ADR even in the absence of ZnCl_2_. As additional control, RKO cells were also treated with ZnCl_2_ alone that did not allow p53 binding to DNA promoters (Fig. 3a, *right panel*, lane 6). Next, *in vivo* analyses of mRNA levels upon dose–response ADR treatment, with or without ZnCl_2_, shows that ADR alone induced the upregulation of p53 apoptotic target genes (here, Noxa and Puma) starting at 0.5 and 1 μg/ml, respectively for RKO and HCT116 cells, while ZnCl_2_ co-treatment induced p53 target gene expression starting at 0.1 μg/ml ADR (Fig. 3b), that is, by lowering the ADR dose 5 to 10 times, depending on cell sensitivity. Densitometric analyses show that the induction of these genes was basically always higher in the presence of ZnCl_2_, and the statistical significance was indeed reached at three ADR dose levels (0.1, 0.2 and 0.5 μg/ml) and for both cell lines, while this difference faded at ADR 1 μg/ml (Fig. 3c), suggesting that functionally speaking these two experimental conditions become almost undistinguishable when higher doses of ADR were used (with some cell line-specific features). Additionally, it is interesting to note that for both cell lines, ADR 0.5 μg/ml with ZnCl_2_ was about like ADR 1 μg/ml without ZnCl_2_ (or even stronger in HCT116); this outcome seems to be promising in the perspective to optimize this treatment for a possible clinical application.

These results demonstrate that zinc supplementation improves wtp53/DNA binding and transcriptional activities in response to low-dose ADR with respect to ADR alone.

### ZnCl_2_ improves the low-dose ADR-induced tumor regression *in vivo*

**Fig. 4:**
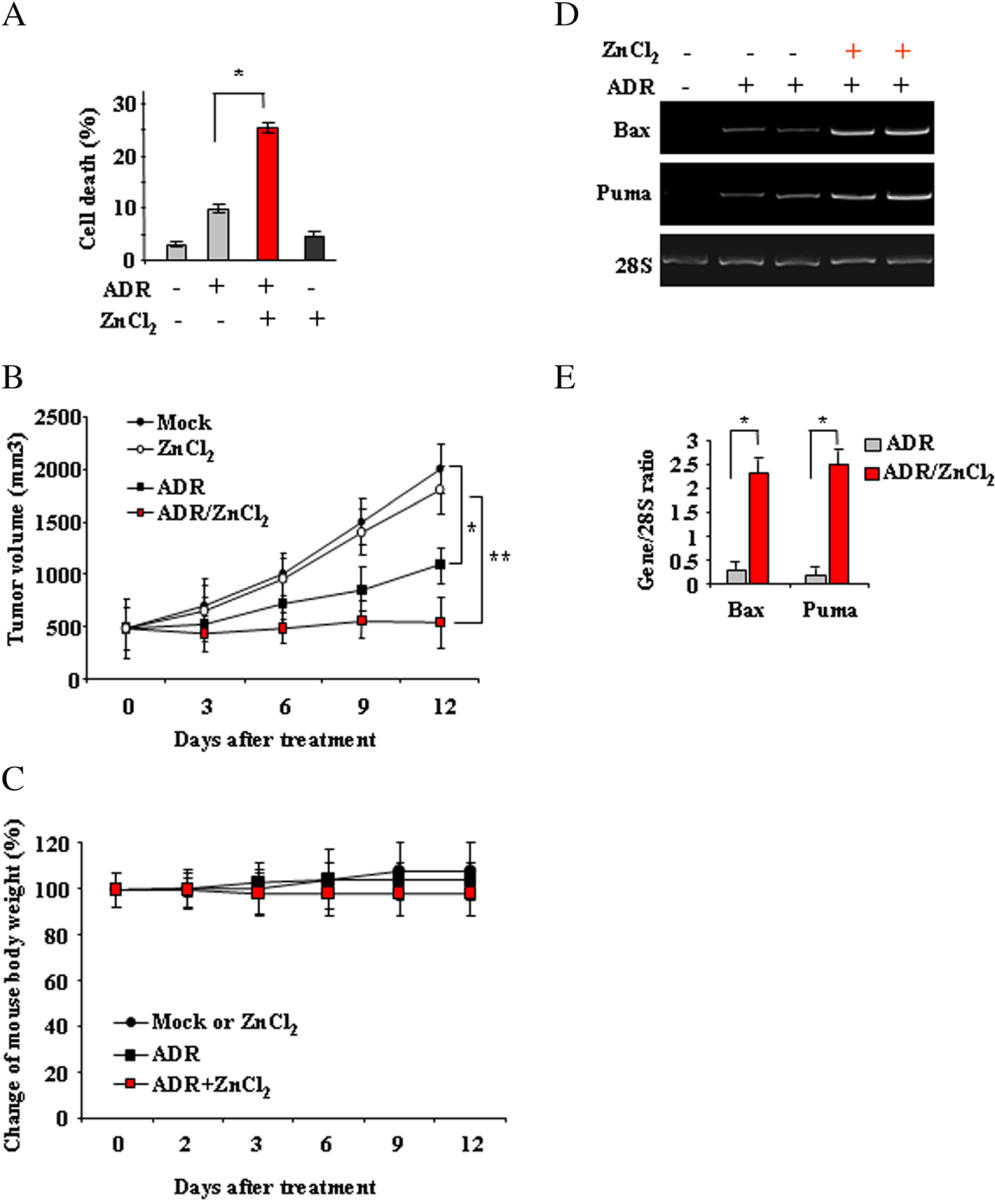
Zinc supplementation improves the antitumor effects of low-dose ADR. **a** RKO cells (2x10^5^) were plated at subconfluence in 60-mm Petri dish and the day after treated with ADR (0.1 μg/ml) and ZnCl_2_ (100 μM) alone or in combination for 24 h. Cell death was measured by trypan blue exclusion assay and expressed as percentage ± SD of three independent experiments performed in duplicate. Paired student’s *t* test was used for the statistical analysis of cell death percentages in the presence of ADR alone or in combination with ZnCl_2_ (*: *P* < 0.01). **b** RKO cells were injected i.m. into the right flanks of nude mice. Drug and ZnCl_2_ treatments were started when the tumors became palpable, and the size of tumors was measured twice a week and plotted as the average tumor volumes *versus* number of days post-treatment. Paired student’s *t* test was used for the statistical analysis of tumor volumes in the presence of ADR alone (*black square*) or in combination with ZnCl_2_ (*red square*) (*: *P* < 0.01). **c** Percentage change of mouse body weight measured throughout the experiment. **d** Tumors were explanted at day 12 after treatments and used for total mRNA extraction and RT-PCR analysis of p53 target genes. 28S was used as a control for efficiency of RNA extraction and transcription. RNA samples from explanted tumors show respectively one Mock tumor, two ADR-treated tumors and two ADR/ZnCl_2_-treated tumors. One representative experiment, out of two independent experiments generating similar results, is shown here. **e** Densitometric analysis of gene expression was plotted as expression ratio to 28S. The data represent the mean of two independent experiments ± S.D. Paired student’s *t* test was used for the statistical analysis of the normalized gene expression (Gene/28S ratios) at different ADR doses, with or without ZnCl_2_ (*: *P* < 0.01)

To measure the biological effect of zinc supplementation to low-dose ADR we first analysed cell viability by trypan blue staining. We found that the negligible effect of low-dose ADR on cell death was significantly increased by ZnCl_2_ (Fig. 4a). We next measured induction of apoptosis by western blot analysis of PARP cleavage. We found that ADR alone induced PARP cleavage starting at 1 μg/ml in HCT116 cells (Fig. 1b), concomitant to p53 expression; interestingly, ZnCl_2_ co-treatment induced PARP cleavage at ADR concentration as low as 0.1 μg/ml (Fig. 1b). Similar results were observed in RKO cells (data not shown). To evaluate whether the administration of ZnCl_2_ is able to improve the efficacy of ADR on tumor growth *in vivo*, we generated tumor xenografts in athymic nude mice by injecting RKO cells. We previously reported that ZnCl_2_ could enhance the antitumor effect of ADR used at 10 mg/Kg body weight in RKO xenografts [29], here, we attempted to perform similar experiments by lowering ten times the amount of ADR used (that is, 1 mg/kg body weight); the aim was to test *in vivo* the results obtained here in the *in vitro* experiments. Groups of CD-1 nude mice were treated with ADR and ZnCl_2_ alone or in combination and tumor growth analysed for about two weeks after the beginning of treatments. Although tumors treated with low-dose ADR alone displayed significant reduction of tumor volume over the course of 2 weeks treatment (ADR *versus* Mock or ZnCl_2_: *P = 0.05, day 12), (Fig. 4b), a significant greater delay of tumor growth was observed in the combination of ZnCl_2_ and low-dose ADR group (ADR/ZnCl_2_*versus* ADR: **P = 0.01) (Fig. 4b). The weight of the mice was also measured twice a week to highlight possible toxicity and the change of mouse body weight was plotted in Fig. 4c. We observed a slight reduction of body weight during the treatments which was comparable between the ADR and ADR/ZnCl_2_ groups (Fig. 4c). Tumors were harvested by day 12 after the beginning of treatments and mRNA extracted for p53 target gene expression by RT-PCR and densitometric analyses. The results show that p53 apoptotic target genes, including Puma and Bax, were slightly induced by low-dose ADR and markedly increased by ZnCl_2_ supplementation (Fig. 4d), as evidenced by densitometric analyses of the normalized gene expression (Fig. 4e). Taken together, these data show that *in vivo* ZnCl_2_ supplementation markedly improved tumor regression in response to low-dose ADR in part due to increasing wtp53 activity, in agreement with the *in vitro* data.

## Discussion

The results of this study highlight the beneficial effect of zinc supplementation in anticancer therapeutic regimens of wtp53-carrying colon cancer cells. Interestingly, zinc-induced p53 activation allowed reducing of about ten times the amount of ADR used with still efficient cytotoxic antitumor effect. The final biological outcome was improved tumor response to low-dose ADR *in vitro* and *in vivo*.

P53 activation is a big matter in cancer field involving the discovery of the mechanisms of p53 activation/inactivation and the development of drug targeted molecules for the achievement of efficient response to therapies [5, 17]. The p53 oncosuppressor activities, such as cell cycle arrest, apoptosis, or senescence, are achieved through the control of p53-mediated transcription of target genes involved in cell-cycle arrest, senescence, (e.g., p21/WAF1/Cip1) or apoptosis (e.g., Puma, Noxa, Bax, p53AIP1etc.) [30]. Activation of p53 can be modulated at several levels such as increase of the p53 protein stabilization with subsequent protein phosphorylation, switch to a folded conformation suitable for binding to canonical DNA promoters, and efficient nuclear translocation for transactivation function [31]. The p53 protein is kept at a low concentration through rapid degradation by its natural inhibitor MDM2 [32] and the stabilization of a functional p53 is reached under precise circumstances such as DNA damage, deprivation of growth factors, oncogene activation, etc. [5]. Here we found that ZnCl_2_ supplementation induced p53 stabilization even in the absence of genotoxic damage (as shown by lack of γH2AX phosphorylation) and, subsequently, p53 activity in combination with low-dose ADR that used alone did not achieve such effects. Mechanistically, zinc supplementation reduced the p53 binding to MDM2, in agreement with previous studies showing that zinc inhibits proteasome-dependent protein degradation [33] and neutralizes the ubiquitine ligase activity of p53 natural inhibitor MDM2 [27]. This finding was further supported here by phosphorylation at N-terminal Ser15 and Ser46 (Fig. 1c) that has been shown to contribute to p53 stability by preventing MDM2 from degrading it [25, 26]. To the best or our knowledge this is the first time that ZnCl_2_ was employed in wtp53-carrying cancer cells to test wtp53 activation in combination with low-dose anticancer drugs. Indeed, recent studies also from us, taking advantage of seminal data of the past years, exploited the effect of zinc ion to target the misfolding of mutant p53 proteins for reactivation of wtp53 oncosuppressive activity [11–16]. Although p53 mutation is usually a late event in colon cancer progression [34], its protein inactivation may be achieved by different mechanisms. Chelation experiments carried out in 1993 first demonstrated that p53 is a zinc-dependent metalloprotein [35] and that zinc is necessary for p53 site-specific DNA binding and proper transcriptional activation [7, 11]. Many studies report that cellular zinc deficiency can be achieved by different conditions such as age, insufficient dietetic intake [36], or by alteration of metallothioneins (MTs). MT is a class of cysteine-rich, metal binding proteins that controls the intracellular distribution of zinc, acts as a potent chelator to remove zinc from wtp53 and modulate p53 conformation and transcriptional activity [8, 10]. Increased expression of MT can be found in various human tumors including colon, and some evidence also supports a role for MT in acquired drug-resistance [37] and in inhibition of wtp53 activity [13, 38]. These findings suggest that zinc might be a valuable molecule to activate wtp53 in cancer cells also in the absence of *TP53* gene mutations. Several studies have shown that p53 reconstitution results in regression of various types of tumors [39, 40] while normal tissues are not significantly affected by genetic p53 re-establishment [41, 42]. By contrast, it appears that the environment of tumor cells supports p53-mediated growth suppression, resulting in a more drastic response induced by p53.

An interesting issue highlighted by our results is the possibility to reduce the amount of chemotherapeutic drugs while maintaining efficient cytotoxic effects. Indeed, the use of some broad spectrum chemotherapeutic drugs such as Adriamycin has been limited in oncologic practice by its overwhelming and harmful dose-dependent effects leading to irreversible and often fatal drug-induced congestive heart failure [22, 23]. The possibility to reduce the amount of ADR is therefore an important goal and the results that we have shown here suggest that the combination with zinc supplementation could be explored in clinical practice to reduce the amount of ADR in the attempt to improve the treatment of CRC as well as patient survival. Indeed, the fact that ZnCl_2_ in combination with low-dose ADR did not negatively affect the good health of animals in the *in vivo* experiment (as shown by body weight analysis), supports the use of such combination treatment in order to reduce toxic effects in sites different than the tumor. More in-depth studies will be needed to better clarify this topic. In addition, whether the reduction of the amount of drugs might be reached by ZnCl_2_ in combination with chemotherapeutic agents different than ADR needs to be addressed. Another issue to keep in mind is that there are many zinc-associated enzymes and transcription factors in cells and that the zinc supplementation might affect many biological processes *in vivo*. In this regard, we previously described the positive effect of zinc in modulating several pathways involved in chemoresistance, angiogenesis, tumor migration and immunological response [14, 43–46], and that zinc complex, orally administrated, preferentially localizes into the tumor site [43], supporting the positive effect of such supplementation in anticancer therapies.

In conclusion, the present study provides insight into the clinically translatable approach of a combination strategy using low-dose ADR and ZnCl_2_ in wtp53-carrying cancer cells with the double result of improving wtp53 activity and reducing the dose of chemotherapy for cytotoxic antitumor purposes.

## Abbreviations

ADR: Adriamycin
ChIP: chromatin immunoprecipitation
DBD: DNA binding domain
DDR: DNA damage response
DSB: double strand brakes
RT-PCR: reverse transcription polymerase chain reaction
SD: standard deviation
wt p53: wild-type p53
ZnCl_2_: zinc chloride
